# Supplementary material for: Sensory cortex wiring requires preselection of short- and long-range projection neurons through an Egr-Foxg1-COUP-TFI network
Source: Nat Commun. 2019 Aug 8;10:3581. doi: 10.1038/s41467-019-11043-w (PMC6687716; doi:10.1038/s41467-019-11043-w)
Supplement: Supplementary file 1 — Supplementary Information [file 41467_2019_11043_MOESM1_ESM.pdf]

SUPPLEMENTARY INFORMATION

Sensory cortex wiring requires preselection of short- and long-range projection neurons through an Egr-Foxg1-COUP-TFI network

Hou et al.

Supplementary Figure 1
Supplementary Figure 2
Supplementary Figure 3
Supplementary Figure 4
Supplementary Figure 5
Supplementary Figure 6
Supplementary Figure 7
Supplementary Figure 8
Supplementary Figure 9
Supplementary Figure 10
Supplementary Figure 11

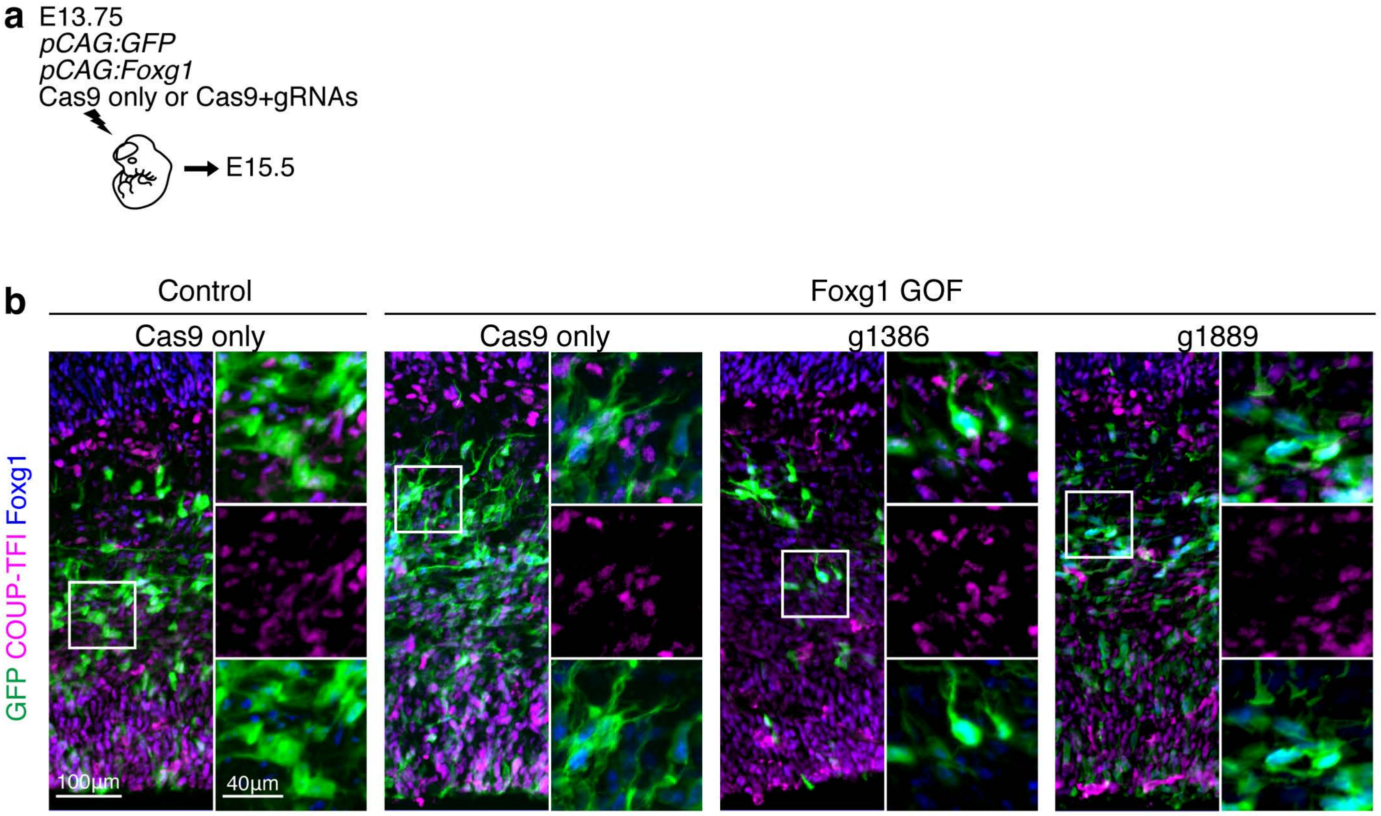
a
E13.75
pCAG:GFP
pCAG:Foxg1
Cas9 only or Cas9+gRNAs
E15.5
b
Control
Foxg1 GOF
Cas9 only
Cas9 only
g1386
g1889
GFP COUP-TFI Foxg1
100µm
40µm

Supplemental Figure 1. The effect of single gRNA introduction upon Foxg1 overexpression

a Schematic diagram of i*n utero* electroporation. Brains were introduced with *pCAG:GFP* and *pCAG:*empty (Control) or *pCAG:GFP* and *pCAG:Foxg1* (Foxg1 GOF).
b Foxg1 (blue), GFP (green) and COUP-TFI (red) staining in E15.5 cortices. Closed arrowheads indicate GFP cells with COUP-TFI expression and open arrowheads indicate GFP cells without COUP-TFI expression.

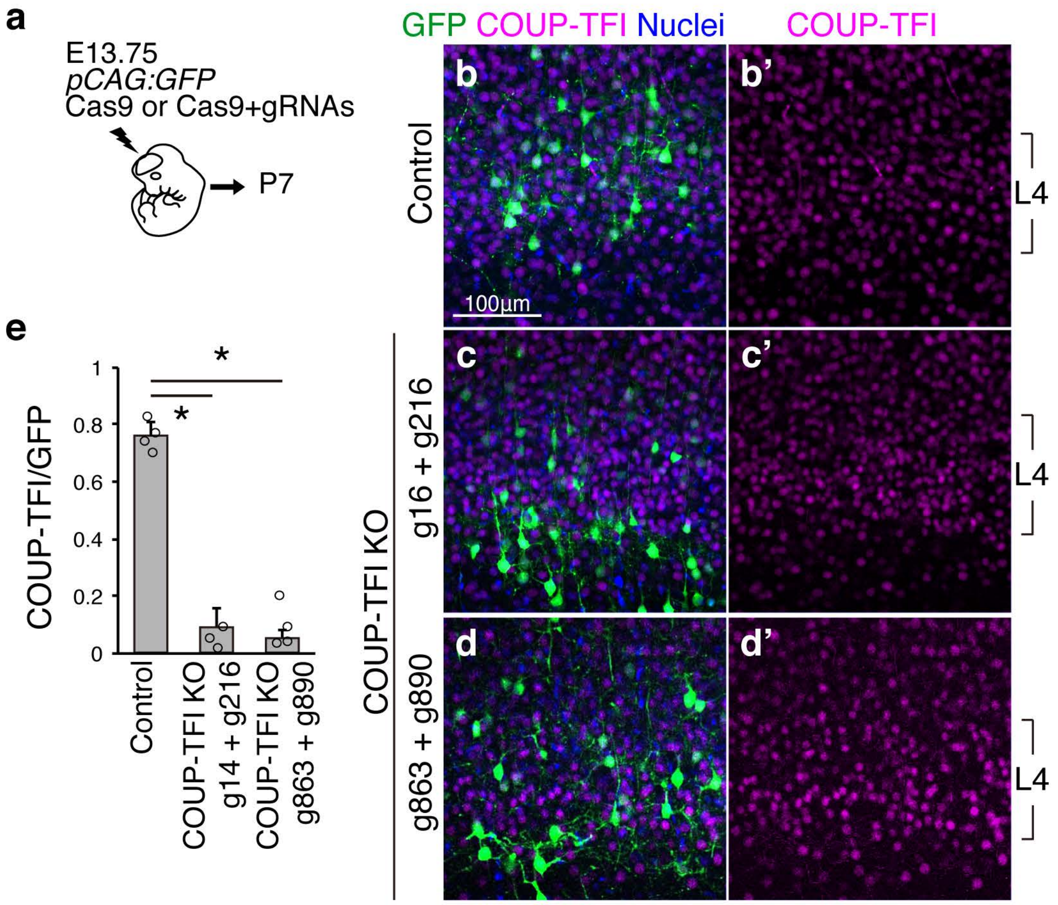
a
E13.75
pCAG:GFP
Cas9 or Cas9+gRNAs
P7
GFP COUP-TFI Nuclei
COUP-TFI
b
b'
c
c'
d
d'
Control
COUP-TFI KO
g16 + g216
g863 + g890
L4
100µm
e
COUP-TFI/GFP
0
0.2
0.4
0.6
0.8
1
*
*
Control
COUP-TFI KO g14 + g216
COUP-TFI KO g863 + g890

Supplemental Figure 2. The efficiency of *COUP-TFI* loss-of-function

a Schematic diagram of *in utero* electroporation. Brains were introduced with *pCAG:GFP* and UbC:Cas9 (Control) or *pCAG:GFP*, UbC:Cas9 and gRNAs (COUP-TFI KO). Combination of gRNAs are as indicated.
b-d' Immunostaining of GFP (green), COUP-TFI (red) and Hoechst 33342 (blue) in P7 cortices.
e Quantitative analysis of the percentage (±SEM) of GFP cells that express COUP-TFI.
* indicates P value < 0.05 by Student's t-test. Source data are provided as a Source Data file.

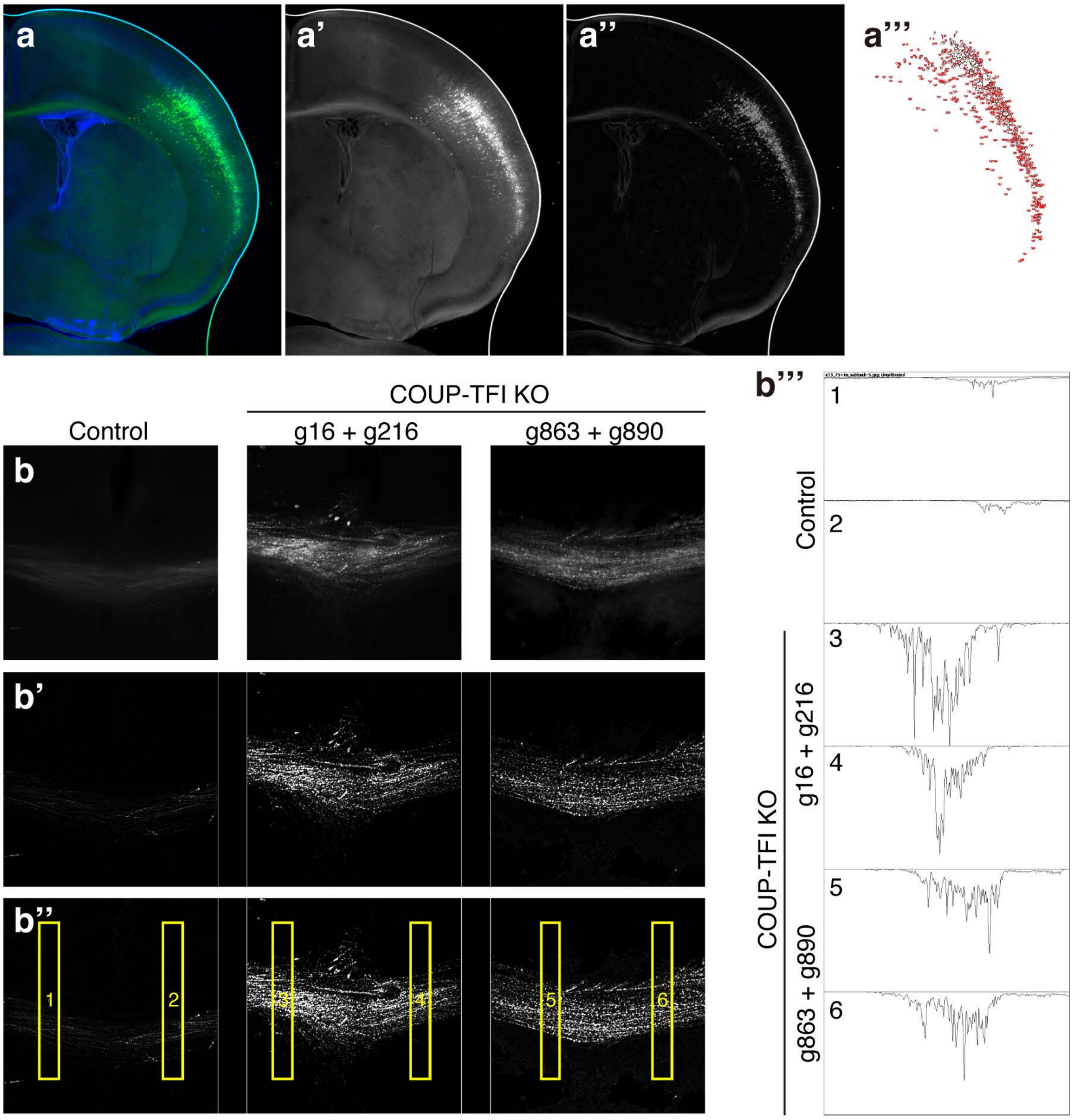
a
a'
a''
a'''
COUP-TFI KO
Control
g16 + g216
g863 + g890
b
b'
b''
1
2
3
4
5
6
b'''
Control
COUP-TFI KO
g16 + g216
g863 + g890

Supplemental Figure 3. The quantification procedure of callosal axons in the corpus callosum

a-a''' The procedure for image processing for cell counting. The GFP signal was extracted (a'). After background subtraction (a''), the number of cells was counted by Analyze Particles function in ImageJ software (a''', see *Methods* for details).
b-b''' The procedure for image processing for GFP signal intensity in the corpus callosum. The GFP signal was extracted from Figure 3i', j' and k' (b). After background subtraction (b'), paired regions in each condition were selected at similar positions (b''). The signal was plotted for measuring GFP intensity (b''', see *Methods* for details).

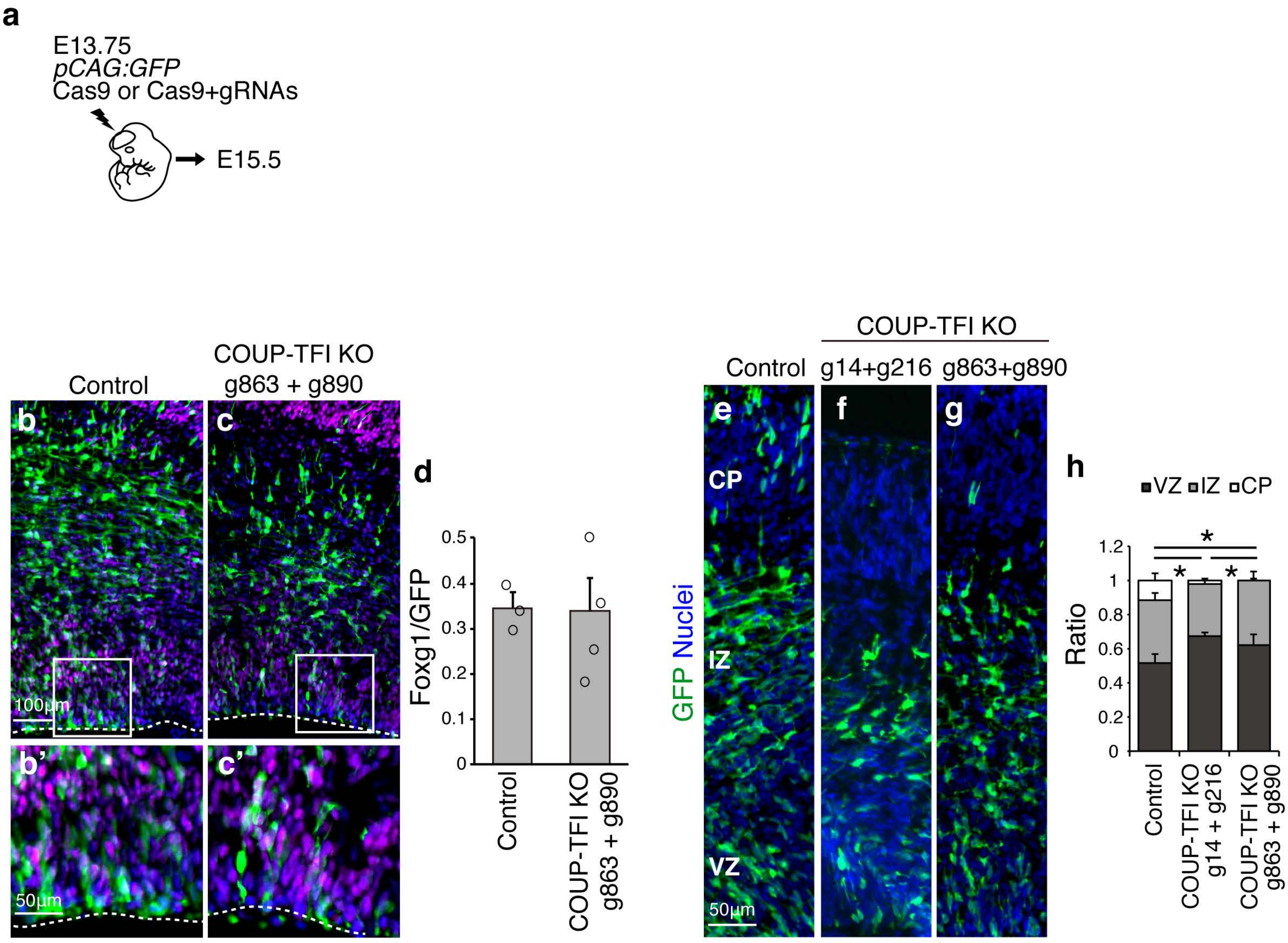
a
E13.75
pCAG:GFP
Cas9 or Cas9+gRNAs
E15.5
Control
COUP-TFI KO
g863 + g890
b
c
b'
c'
GFP Foxg1 Nuclei
100µm
50µm
d
Foxg1/GFP
0.5
0.4
0.3
0.2
0.1
0
Control
COUP-TFI KO
g863 + g890
COUP-TFI KO
Control
g14+g216
g863+g890
e
f
g
CP
IZ
VZ
GFP Nuclei
50µm
h
VZ IZ CP
Ratio
1.2
1
0.8
0.6
0.4
0.2
0
*
*
*
Control
COUP-TFI KO
g14 + g216
COUP-TFI KO
g863 + g890

Supplemental Figure 4. The effect of *COUP-TFI* loss-of-function on neuronal migration

a Schematic diagram of *in utero* electroporation. Brains were introduced with *pCAG:GFP* and *pUbC:Cas9* only (Control) or *pCAG:GFP*, *pUbC:Cas9* and gRNAs (COUP-TFI KO). Combination of gRNAs are as indicated.
b-c' Immunostaining of GFP (green), Foxg1 (red) and Hoechst 33342 (blue) in E15.5 cortices. Dashed lines indicate the ventricular surface.
d Quantitative analysis of the percentage (±SEM) of GFP cells that express Foxg1.
e-g Immunostaining of GFP (green) and Hoechst 33342 (blue) in E15.5 cortices.
h Quantitative analysis of the distribution of GFP cells.
* indicates P value < 0.05 by two-way ANOVA. Source data are provided as a Source Data file.

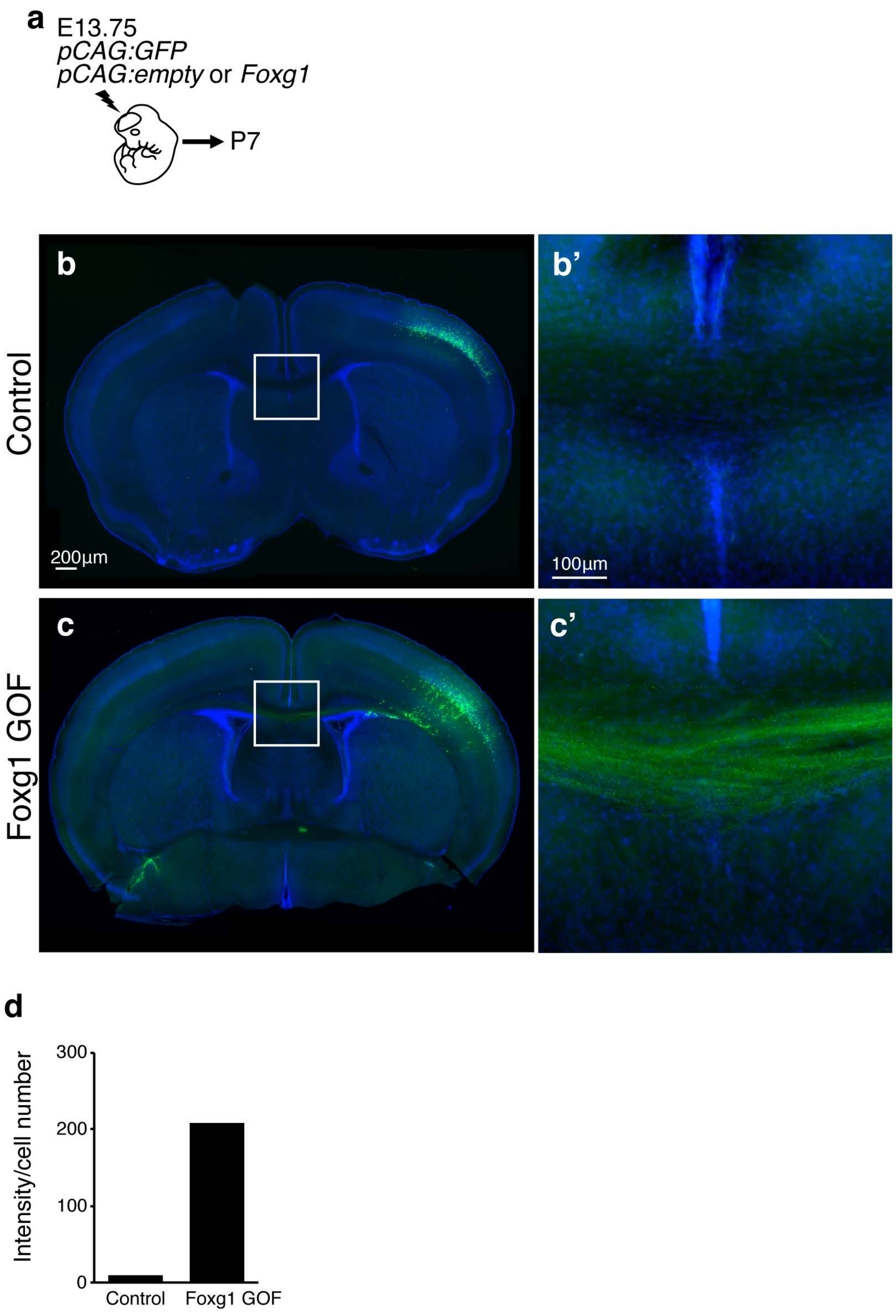
a
E13.75
pCAG:GFP
pCAG:empty or Foxg1
P7
b
b'
Control
200µm
100µm
c
c'
Foxg1 GOF
d
Intensity/cell number
300
200
100
0
Control
Foxg1 GOF

Supplemental Figure 5. Callosal axon analysis of *Foxg1* gain-of-function cortices

a Schematic diagram of *in utero* electroporation. Brains were introduced with *pCAG:GFP* and *pCAG:empty* (Control) or *pCAG:GFP* and *pCAG:Foxg1* (Foxg1 GOF).
b-c' Immunostaining of GFP (green) and Hoechst 33342 (blue) in P7 cortices. b'-c' demarcate enlarged view of boxed regions shown in b-c.
d Quantitative analysis of the GFP signal intensity in the corpus callosum normalized to GFP-positive cell number (see *Methods* for details).

Supplemental_Fig. 6

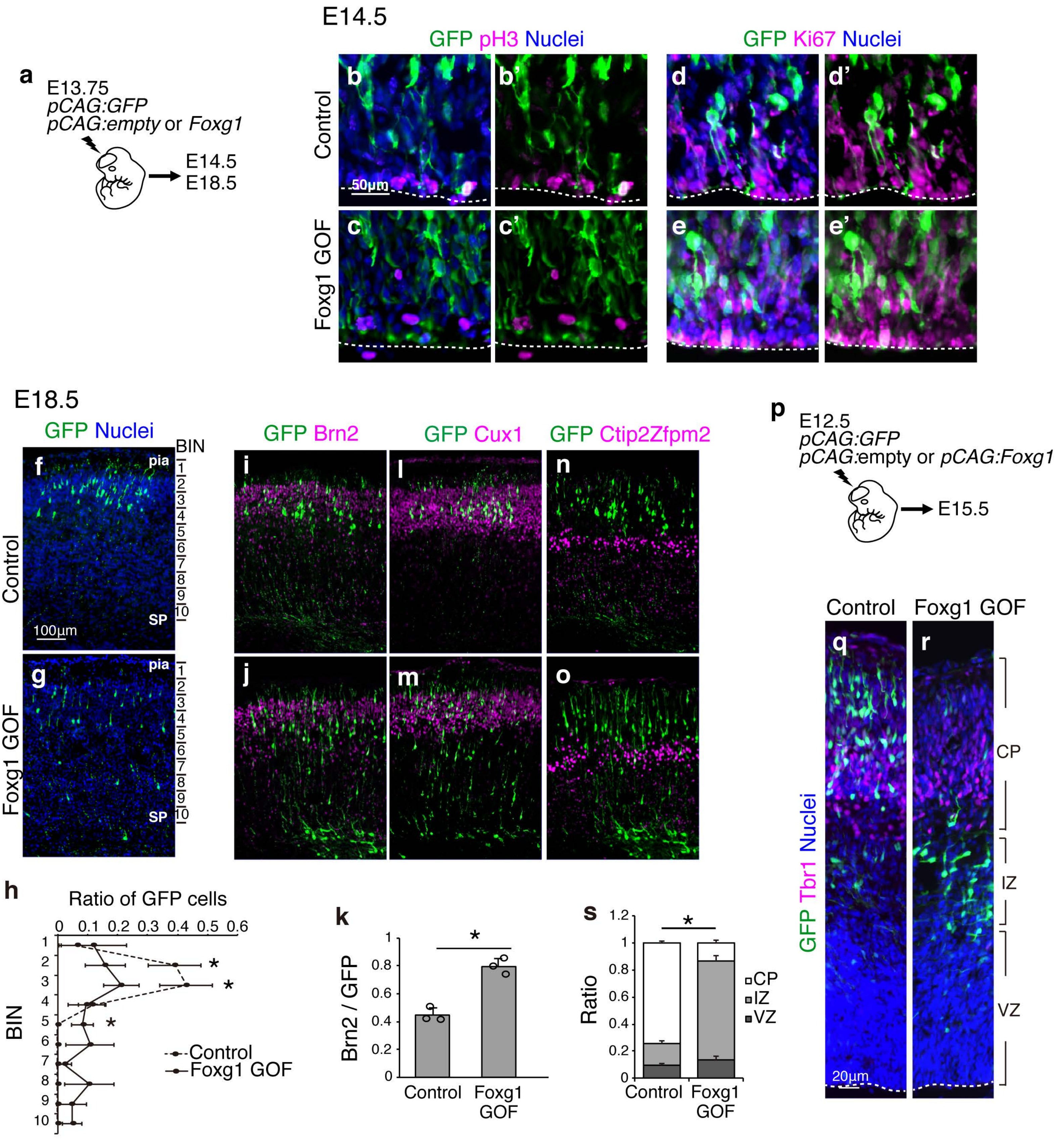

Supplemental Figure 6. ***Foxg1*** **gain-of-function analysis at distinct developmental timepoints.**

a Schematic diagram of *in utero* electroporation. Brains were introduced with *pCAG:GFP* and *pCAG:*empty (Control) or *pCAG:GFP* and *pCAG:Foxg1* (Foxg1 GOF).
b-e' Immunostaining for GFP (green), Hoechst 33342 (blue) and M-phase marker pH3 (red) (b-c') or proliferation marker Ki67 (red) (d-e') in E14.5 cortices. Dashed lines indicate the ventricular surface.
f-g Immunostaining for GFP (green) and Hoechst 33342 (blue) of E18.5 cortex. Cortical plate is divided into 10 BINs from the pia to the subplate. SP, subplate.
h Quantitative analysis of the distribution of GFP cells in E18.5 cortices.
i-o Immonostaining of GFP (green) and Brn2 (i-j) or Cux1 (red) (l-m) or Ctip2/Zfpm2 (n-o) in E18.5 cortices. (k) Quantitative analysis of the percentage (±SEM) of GFP cells that express Brn2.
p Schematic diagram of *in utero* electroporation. Brains were introduced with *pCAG:GFP* and *pCAG:*empty (Control) or *pCAG:GFP* and *pCAG:Foxg1* (Foxg1 GOF).
q-r Immunostaining for GFP (green), Hoechst 33342 (blue) and layer 6 marker Tbr1 (red) in E15.5 cortices. Dashed lines indicate the ventricular surface.
s Quantitative analysis of the distribution of GFP cells in E15.5 cortices. VZ, ventricular zone. IZ, intermediate zone. CP, cortical plate.
* indicates P value < 0.05 by Student's t-test (h and k) or two-way ANOVA (s). Source data are provided as a Source Data file.

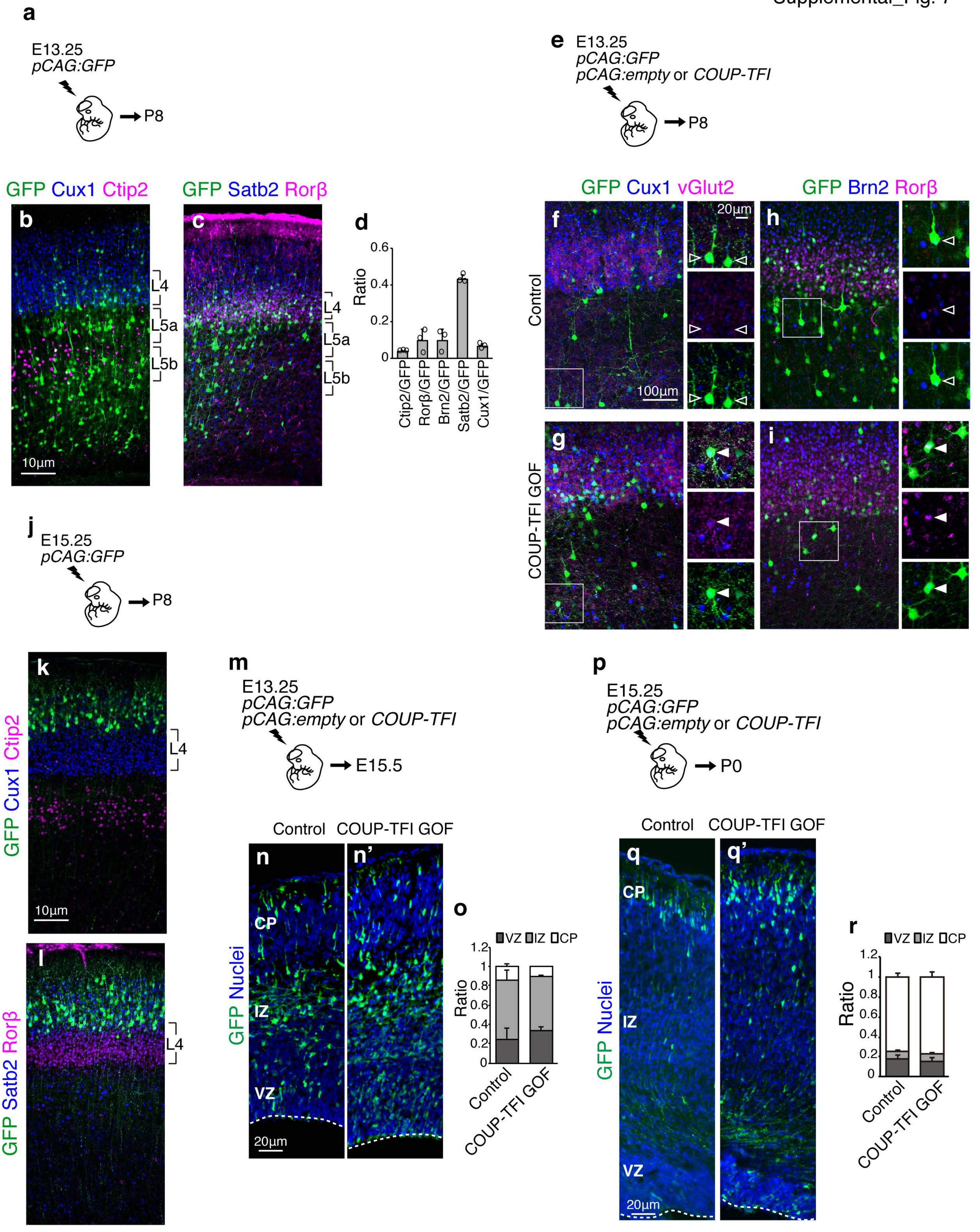
a
E13.25
pCAG:GFP
P8
GFP Cux1 Ctip2
b
L4
L5a
L5b
10µm
GFP Satb2 Rorβ
c
L4
L5a
L5b
d
Ratio
0.6
0.4
0.2
0
Ctip2/GFP
Rorβ/GFP
Brn2/GFP
Satb2/GFP
Cux1/GFP
e
E13.25
pCAG:GFP
pCAG:empty or COUP-TFI
P8
GFP Cux1 vGlut2
GFP Brn2 Rorβ
f
20µm
h
Control
100µm
g
i
COUP-TFI GOF
j
E15.25
pCAG:GFP
P8
k
GFP Cux1 Ctip2
L4
10µm
l
GFP Satb2 Rorβ
L4
m
E13.25
pCAG:GFP
pCAG:empty or COUP-TFI
E15.5
Control
COUP-TFI GOF
n
n'
CP
IZ
VZ
20µm
GFP Nuclei
o
VZ IZ CP
1.2
1
0.8
0.6
0.4
0.2
0
Ratio
Control
COUP-TFI GOF
p
E15.25
pCAG:GFP
pCAG:empty or COUP-TFI
P0
Control
COUP-TFI GOF
q
q'
CP
IZ
VZ
20µm
GFP Nuclei
r
VZ IZ CP
1.2
1
0.8
0.6
0.4
0.2
0
Ratio
Control
COUP-TFI GOF

Supplemental Figure 7. ***COUP-TFI*** **gain-of-function analysis at distinct developmental timepoints.**

a Schematic diagram of *in utero* electroporation.
b-c Immunostaining of P8 cortices for GFP (green), Cux1 (blue) and Ctip2 (red) (b) or GFP (green), Satb2 (blue) and Rorβ (red) (c).
d Quantitative analysis of marker gene expression in GFP cells labeled at E13.25.
e Schematic diagram of *in utero* electroporation. Brains were introduced with *pCAG:GFP* and *pCAG:*empty (Control) or *pCAG:GFP* and *pCAG:COUP-TFI* (COUP-TFI GOF). Closed arrowheads indicate GFP cells with vGlut2 or Rorβ expression and open arrowheads indicate GFP cells without vGlut2 or Rorβ expression.
f-i Double immunohistochemistry of P8 cortices for GFP (green) with vGlut2 (red) and Cux1 (blue) (f-g), or Rorβ (red) and Brn2 (blue) (h-i).
j Schematic diagram of *in utero* electroporation.
k-l Immunostaining of P8 cortices for GFP (green), Cux1 (blue) and Ctip2 (red) (k) or GFP (green), Satb2 (blue) and Rorβ (red) (l).
m Schematic diagram of *in utero* electroporation. Brains were introduced with *pCAG:GFP* and *pCAG:*empty (Control) or *pCAG:GFP* and *pCAG:COUP-TFI* (COUP-TFI GOF).
n-n' Immunostaining of E15.5 cortices for GFP (green) and Hoechst 33342 (blue). Dashed lines indicate ventricular surface. VZ, ventricular zone. IZ, intermediate zone. CP, cortical plate.
o Quantitative analysis of the percentage (±SEM) of GFP position in E15.5 cortices.
p Schematic diagram of *in utero* electroporation. Brains were introduced with *pCAG:GFP* and *pCAG:*empty (Control) or *pCAG:GFP* and *pCAG:COUP-TFI* (COUP-TFI GOF).
q-q' Immunostaining for GFP (green) and Hoechst 33342 (blue) in P0 cortices.
r Quantitative analysis of the percentage (±SEM) of GFP position in P0 cortices. Dashed lines indicate ventricular surface. VZ, ventricular zone. IZ, intermediate zone. CP, cortical plate.

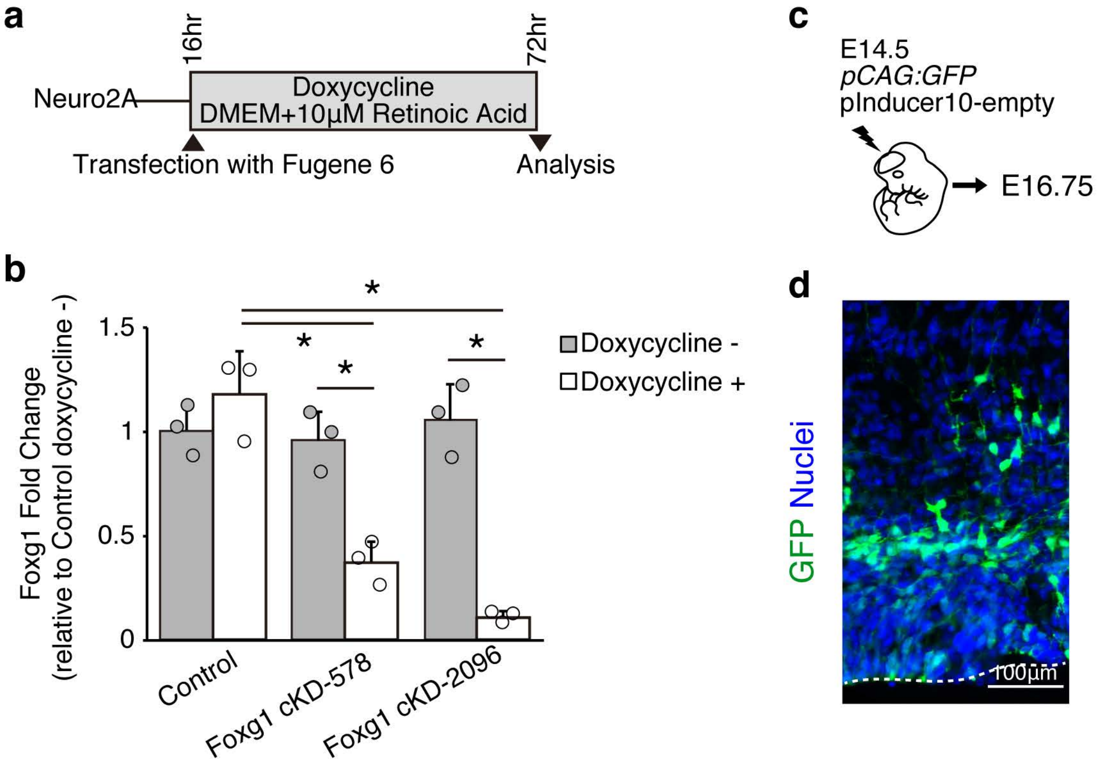
a
16hr
72hr
Neuro2A
Doxycycline
DMEM+10μM Retinoic Acid
Transfection with Fugene 6
Analysis
b
Foxg1 Fold Change
(relative to Control doxycycline -)
1.5
1
0.5
0
Control
Foxg1 cKD-578
Foxg1 cKD-2096
Doxycycline -
Doxycycline +
c
E14.5
pCAG:GFP
pInducer10-empty
E16.75
d
GFP Nuclei
100μm

Supplemental Figure 8. Validation of conditional *Foxg1* knockdown using the *pInducer* system.

a Schematic diagram showing transfection of plasmids carrying *pInducer10*-empty, *pInducer10-shFoxg1-578*, or *pInducer10-shFoxg1-2096* into Neuro2A cells. 16hr after plating, medium was changed into differentiation medium containing 2μg/ml doxycycline and samples were collected at 72hr.
b qRT-PCR analysis of *Foxg1* mRNA expression (mean ± SEM). Cells were introduced with *pInducer10*-empty (Control), *pInducer10-shFoxg1-578* (Foxg1 cKD-578) or *pInducer10-shFoxg1-2096* (Foxg1 cKD-2096). Cells with or without doxycycline treatment are indicated as doxycycline + and doxycycline -, respectively. Values indicated relative Foxg1 expression fold change to doxycycline- Control. βActin was used as an internal control for normalization.
c Schematic diagram of *in utero* electroporation.
d Immunostaining of E16.75 cortices for GFP (green) and Hoechst 33342 (blue). Dashed lines indicate the ventricular surface.
* indicates P value < 0.05 by Student's t-test. Source data are provided as a Source Data file.

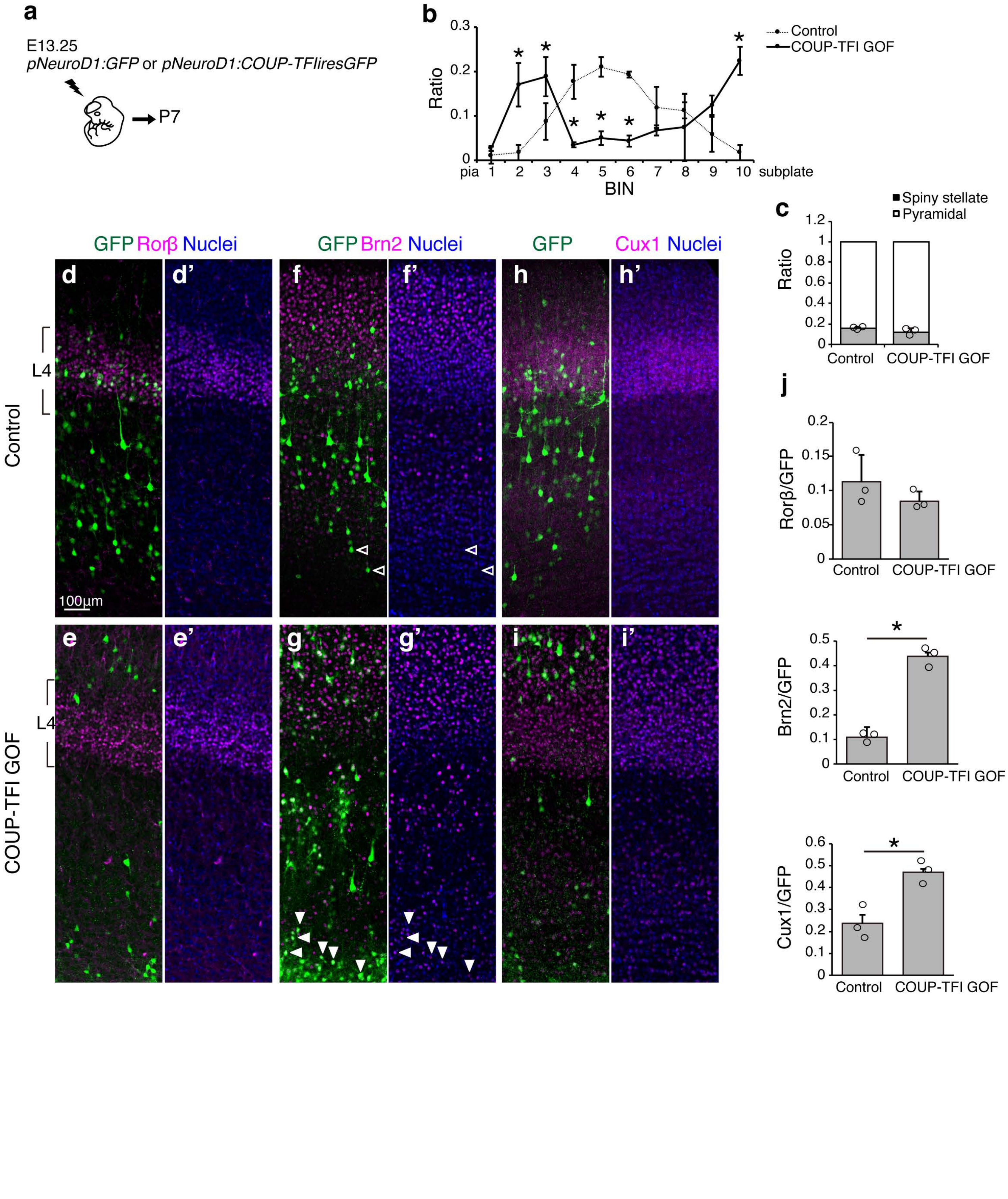
a
E13.25
pNeuroD1:GFP or pNeuroD1:COUP-TFIiresGFP
P7
b
Control
COUP-TFI GOF
Ratio
0.3
0.2
0.1
0
pia 1 2 3 4 5 6 7 8 9 10 subplate
BIN
c
Spiny stellate
Pyramidal
Ratio
1.2
1
0.8
0.6
0.4
0.2
0
Control
COUP-TFI GOF
GFP Rorβ Nuclei
GFP Brn2 Nuclei
GFP Cux1 Nuclei
d
d'
f
f'
h
h'
e
e'
g
g'
i
i'
L4
Control
COUP-TFI GOF
100µm
j
Rorβ/GFP
0.2
0.15
0.1
0.05
0
Control
COUP-TFI GOF
Brn2/GFP
0.5
0.4
0.3
0.2
0.1
0
Control
COUP-TFI GOF
Cux1/GFP
0.6
0.5
0.4
0.3
0.2
0.1
0
Control
COUP-TFI GOF

Supplemental Figure 9. Ectopic COUP-TFI expression driven by the *NeuroD1* promoter fails to convert non-layer 4 cells to layer 4 identity

a Schematic diagram of *in utero* electroporation. Brains were introduced with *pNeuroD1:GFP* (Control) or *pNeuroD1:COUP-TFIiresGFP* (COUP-TFI GOF).
b Quantitative analysis of the distribution of GFP cells in P7 cortices. Cortical plate is divided into 10 BINs from the pia to the subplate.
c Quantitative analysis of the percentage (±SEM) of GFP cells with spiny stellate or pyramidal morphology.
d-i' Double immunohistochemistry of P7 cortices for GFP (green) with Rorβ (red) (d-e'), or Brn2 (red) (f-g'), or Cux1 (red) (h-i'). Closed arrowheads indicate GFP cells with Brn2 expression and open arrowheads indicate GFP cells without Brn2 expression (f-g').
j Quantitative analysis of the percentage (±SEM) of GFP cells that express Rorβ, Brn2 or Cux1.
* indicates P value < 0.05 by Student's t-test. Source data are provided as a Source Data file.

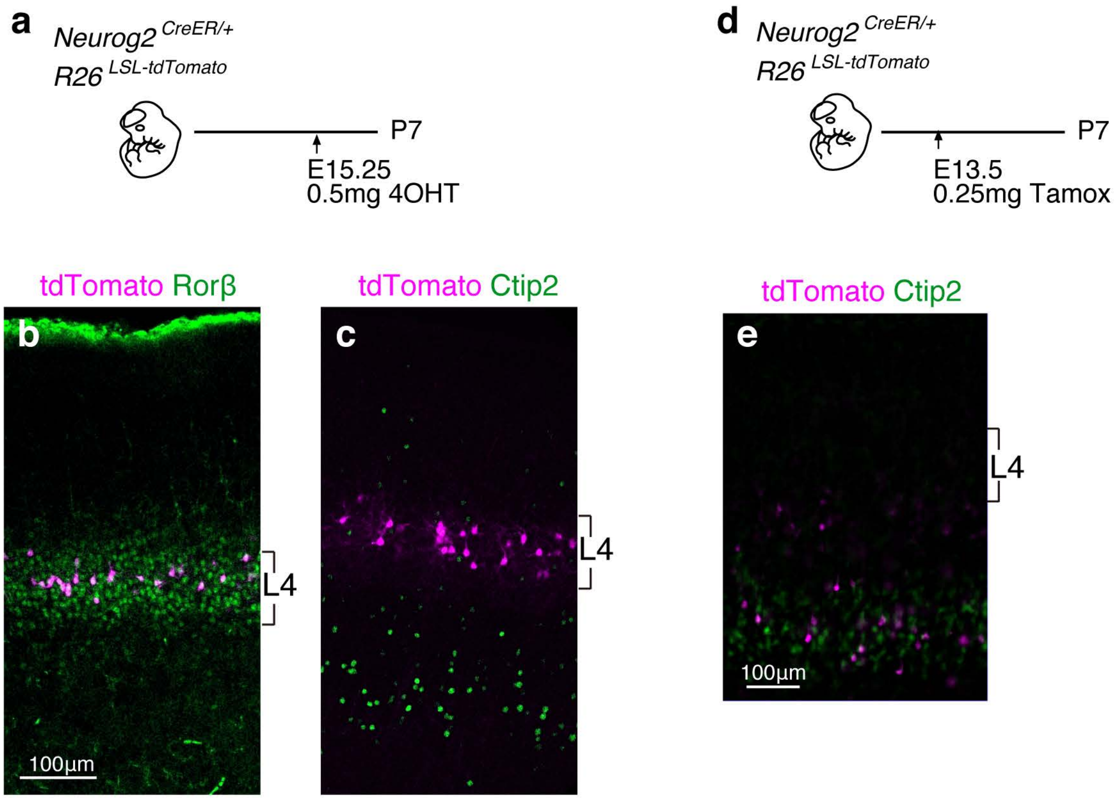
a
Neurog2 CreER/+
R26 LSL-tdTomato
P7
E15.25
0.5mg 4OHT
d
Neurog2 CreER/+
R26 LSL-tdTomato
P7
E13.5
0.25mg Tamox
tdTomato Rorβ
b
L4
100µm
tdTomato Ctip2
c
L4
tdTomato Ctip2
e
L4
100µm

Supplemental Figure 10. Genetic labeling of laminar subtypes using *Neurog2*$^{CreER/+}$ mediated temporal recombination

a Schematic diagram of *Neurog2*$^{CreER/+}$; *R26*$^{LSL\text{-}tdTomato}$ mice with E15.25 4OHT administration. Samples were collected at P7 (b-c).
b-c Immunostaining of P7 cortices with tdTomato (red), Rorβ (green) (b) or Ctip2 (green) (c).
d Schematic diagram of *Neurog2*$^{CreER/+}$; *R26*$^{LSL\text{-}tdTomato}$ mice with E13.5 Tamoxifen administration. Sample were collected at P7 (e).
e Immunostaining of P7 cortices for tdTomato (red) and Ctip2 (green).

a

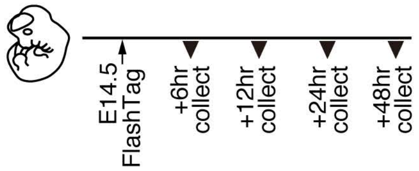
E14.5
FlashTag
+6hr
collect
+12hr
collect
+24hr
collect
+48hr
collect

b

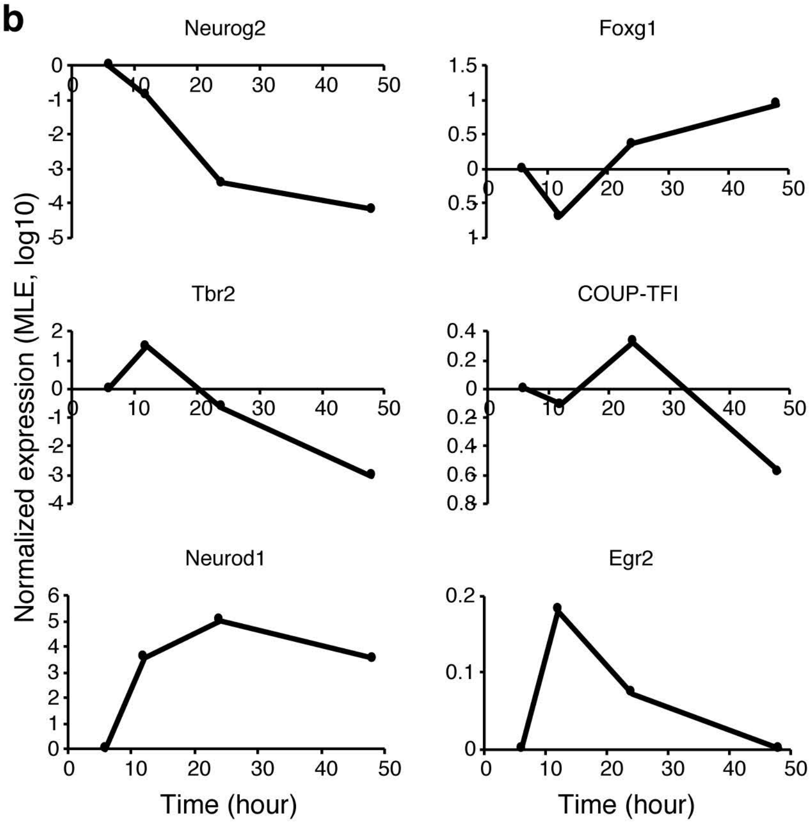
Neurog2
Foxg1
Tbr2
COUP-TFI
Neurod1
Egr2
Normalized expression (MLE, log10)
Time (hour)
Time (hour)

Supplemental Figure 11. Temporal dynamics of neuronal precursor gene expression during corticogenesis

a Schematic diagram of E14.5 FlashTag labeling and sample collection in the transcriptomic analysis (Telley et al. Science 2016)
b The normalized expression value of the indicated genes during neurogenesis (Telley et al. Science 2016)
