# Supplementary material for: Sensory cortex wiring requires preselection of short- and long-range projection neurons through an Egr-Foxg1-COUP-TFI network
Source: Nat Commun. 2019 Aug 8;10:3581. doi: 10.1038/s41467-019-11043-w (PMC6687716; doi:10.1038/s41467-019-11043-w)
Supplement: Supplementary file 3 — Reporting Summary [file 41467_2019_11043_MOESM3_ESM.pdf]

nature research

Corresponding author(s): Carina Hanashima

# Reporting Summary

Nature Research wishes to improve the reproducibility of the work that we publish. This form provides structure for consistency and transparency in reporting. For further information on Nature Research policies, see Authors & Referees and the Editorial Policy Checklist.

## Statistical parameters

When statistical analyses are reported, confirm that the following items are present in the relevant location (e.g. figure legend, table legend, main text, or Methods section).

| n/a | Confirmed | |
|---|---|---|
| ☐ | ☒ | The exact sample size ($n$) for each experimental group/condition, given as a discrete number and unit of measurement |
| ☐ | ☒ | An indication of whether measurements were taken from distinct samples or whether the same sample was measured repeatedly |
| ☐ | ☒ | The statistical test(s) used AND whether they are one- or two-sided<br>*Only common tests should be described solely by name; describe more complex techniques in the Methods section.* |
| ☒ | ☐ | A description of all covariates tested |
| ☐ | ☒ | A description of any assumptions or corrections, such as tests of normality and adjustment for multiple comparisons |
| ☐ | ☒ | A full description of the statistics including central tendency (e.g. means) or other basic estimates (e.g. regression coefficient) AND variation (e.g. standard deviation) or associated estimates of uncertainty (e.g. confidence intervals) |
| ☐ | ☒ | For null hypothesis testing, the test statistic (e.g. $F$, $t$, $r$) with confidence intervals, effect sizes, degrees of freedom and $P$ value noted<br>*Give P values as exact values whenever suitable.* |
| ☒ | ☐ | For Bayesian analysis, information on the choice of priors and Markov chain Monte Carlo settings |
| ☒ | ☐ | For hierarchical and complex designs, identification of the appropriate level for tests and full reporting of outcomes |
| ☒ | ☐ | Estimates of effect sizes (e.g. Cohen's $d$, Pearson's $r$), indicating how they were calculated |
| ☐ | ☒ | Clearly defined error bars<br>*State explicitly what error bars represent (e.g. SD, SE, CI)* |

*Our web collection on statistics for biologists may be useful.*

## Software and code

Policy information about availability of computer code

| | |
|---|---|
| Data collection | No software/code were used for data collection in this study. |
| Data analysis | NeuroLucida 360 (2.70.1, 64bit) from MBF Bioscience was used for reconstruction of GFP cells. For post-sequencing analysis, Illumina HiSeq 1500 for sequencing the reads, bcl2fastq (1.8.4) for base calling, FastQC (0.11.3) for quality control validation, Cutadapt (1.4.1) and Fastz (0.0.14) for TrimTruSeq/Quartz Adapters and low quality bases, BOWTIE (3.3.3) for rRNA/adapter check, HISAT2 (2.0.4) for transcriptome/genome alignment, Cuttfiff (2.2.1) for expression quantification, and EdgeR (3.16.5) for differential expression were used. Gene Cluster 3.0 and Java TreeView 1.1.6r4 were used to create the heatmap of differential gene expression. Ingenuity Pathway Analysis from QIAGEN informatics was used to analyze the upstream regulators. ImageJ from NIH was used for the quantification analysis of callosal axons. |

For manuscripts utilizing custom algorithms or software that are central to the research but not yet described in published literature, software must be made available to editors/reviewers upon request. We strongly encourage code deposition in a community repository (e.g. GitHub). See the Nature Research guidelines for submitting code & software for further information.

## Data

Policy information about availability of data

All manuscripts must include a data availability statement. This statement should provide the following information, where applicable:

- Accession codes, unique identifiers, or web links for publicly available datasets
- A list of figures that have associated raw data
- A description of any restrictions on data availability

TrueSeq Total RNA sequencing data has been deposited to NCBI Sequence Read Archive database with accession number: PRJAN505194.

# Field-specific reporting

Please select the best fit for your research. If you are not sure, read the appropriate sections before making your selection.

☒ Life sciences ☐ Behavioural & social sciences ☐ Ecological, evolutionary & environmental sciences

For a reference copy of the document with all sections, see nature.com/authors/policies/ReportingSummary-flat.pdf

# Life sciences study design

All studies must disclose on these points even when the disclosure is negative.

| | |
|---|---|
| Sample size | For RNA sequencing analysis, we obtained a minimum average of 12M reads for each sample. For Foxg1 GOF, Foxg1 GOF+PBS1 KO, COUP-TFI KO, COUP-TFI GOF, Foxg1 cKD, Egr1 GOF, Egr2 GOF, Egr1 KO, Egr2 KO, Foxg1-promoter KO, NeuroD1:Foxg1 GOF, NeuroD1:COUP-TFI GOF and reporter luciferase studies, a minimum of 3 independent samples were subjected to analysis. |
| Data exclusions | For RNA sequencing analysis, all duplicates were removed in Quality control using FastQC. For other experiments, no data were excluded from the analysis. |
| Replication | Experimental replicates were performed for Foxg1 GOF, Foxg1 GOF+PBS1 KO, COUP-TFI KO, COUP-TFI GOF, Foxg1 cKD, Egr1 GOF, Egr2 GOF, Egr1 KO, Egr2 KO, Foxg1-promoter KO, NeuroD1:Foxg1 GOF and NeuroD1:COUP-TFI GOF with minimum of 2 litters for each condition. ChIP-qPCR analyses were repeated three times independently from cells isolated at different timing producing similar patterns. Results are shown from technical replicates. Reporter luciferase were repeated in three independent wells for each condition. |
| Randomization | Samples were not allocated into experimental groups randomly. However, in each group, samples were collected from multiple litters and without bias. |
| Blinding | Blinding was not relevant to this study to match the experimental samples with appropriate controls. All data was statistically adjusted using corresponding controls. |

# Reporting for specific materials, systems and methods

## Materials & experimental systems

| n/a | Involved in the study |
|---|---|
| ☒ | ☐ Unique biological materials |
| ☐ | ☒ Antibodies |
| ☐ | ☒ Eukaryotic cell lines |
| ☒ | ☐ Palaeontology |
| ☐ | ☒ Animals and other organisms |
| ☒ | ☐ Human research participants |

## Methods

| n/a | Involved in the study |
|---|---|
| ☒ | ☐ ChIP-seq |
| ☒ | ☐ Flow cytometry |
| ☒ | ☐ MRI-based neuroimaging |

## Antibodies

| | |
|---|---|
| Antibodies used | The following antibodies were used in this study: rat anti-GFP (04404-84, NACALAI), chicken anti-GFP (ab13970, Abcam), rabbit anti-BF1/Foxg1 (M227, TaKaRa), mouse anti-COUP-TFI (PP-H8132, Perseus Proteomics), rat anti-Ctip2 (ab18465, Abcam), mouse anti-Rorβ (PP-N7927, Perseus Proteomics), rabbit anti-Cux1/CDP (sc-13024, Santa Cruz), goat anti-Brn2 (SC-6029, Santa Cruz), rabbit anti-Satb2 (ab34735, Abcam), rabbit anti-Zfpm2 (sc-10755, Santa Cruz), guinea pig anti-vGlut2 (ab2251, Millipore), rabbit anti-DsRed (632496, Clontech), mouse anti-Ki67 (550609, BD) and rabbit anti-pH3 (06-570, Millipore). |

Validation

Validations for each antibody were performed by the suppliers and further confirmed using control samples. For each experiment, localization, timing, and signal intensity were validated.
Relevant articles:

Kumamoto T, Toma K, Gunadi, McKenna WL, Kasukawa T, Katzman S, Chen B, Hanashima C. 2013. Foxg1 coordinates the switch from nonradially to radially migrating glutamatergic subtypes in the neocortex through spatiotemporal repression. Cell reports 3: 931-945.

Toma K, Kumamoto T, Hanashima C. 2014. The timing of upper-layer neurogenesis is conferred by sequential derepression and negative feedback from deep-layer neurons. J Neurosci 34: 13259-13276.

Vitali I, Fievre S, Telley L, Oberst P, Bariselli S, Frangeul L, Baumann N, McMahon JJ, Klingler E, Bocchi R et al. 2018. Progenitor Hyperpolarization Regulates the Sequential Generation of Neuronal Subtypes in the Developing Neocortex. Cell 174: 1264-1276 e1215.

## Eukaryotic cell lines

Policy information about cell lines

Cell line source(s)

U87MG was obtained from ATCC.

Authentication

The U87MG cell line was obtained recently from ATCC where the authentication has been performed rigorously.

Mycoplasma contamination

U87MG has not been tested for mycoplasma as the cell line was recently obtained from ATCC and subjected to experiments within 5 passages in this study .

Commonly misidentified lines
(See ICLAC register)

No commonly misidentified cell lines were used.

## Animals and other organisms

Policy information about studies involving animals; ARRIVE guidelines recommended for reporting animal research

Laboratory animals

Mice were housed in the Animal Housing Facility of the RIKEN Center for Developmental Biology (Kobe, Japan) and Waseda University Animal Facility (Tokyo, Japan) following the institutional guidelines.

For Foxg1 GOF, Foxg1 GOF+PBS1 KO, COUP-TFI KO, COUP-TFI GOF, Foxg1 cKD, Egr1 GOF, Egr2 GOF, Egr1 KO, Egr2 KO, Foxg1-promoter KO, NeuroD1:Foxg1 GOF and NeuroD1:COUP-TFI GOF studies, ICR pregnant female mice were obtained from the Animal Housing Facility of the RIKEN Center for Developmental Biology (Kobe, Japan). For RNA sequencing, Neurog2CreER/+ heterozygous male mice were crossed with R26CAG-LSL-tdTomato female mice. Both Neurog2CreER/+ heterozygous male mice and R26CAG-LSL-tdTomato female mice are on a B6 background.

Wild animals

This study did not involve wild animals.

Field-collected samples

This study did not involve field-collected samples.
